# Reversible oxygen-tolerant hydrogenase carried by free-living N_2_-fixing bacteria isolated from the rhizospheres of rice, maize, and wheat

**DOI:** 10.1002/mbo3.37

**Published:** 2012-09-12

**Authors:** Philippe Roumagnac, Pierre Richaud, Mohamed Barakat, Philippe Ortet, Marie-Anne Roncato, Thierry Heulin, Gilles Peltier, Wafa Achouak, Laurent Cournac

**Affiliations:** 1 CIRAD, UMR BGPI, Campus International de Montferrier-BaillarguetF-34398, Montpellier Cedex-5, France; 2 CEA, DSV, IBEB, Laboratoire d'écologie microbienne de la rhizosphère et d'environnements extrêmes, Centre de CadaracheF-13108, Saint-Paul-lez-Durance, France; 3 CNRS, UMR 6191, Biologie Végétale et Microbiologie EnvironnementalesSaint-Paul-lez-Durance, France; 4 Aix-Marseille UniversitéSaint-Paul-lez-Durance, France; 5 CEA, DSV, IBEB, Laboratoire de Bioénergétique et Biotechnologie des Bactéries et MicroalguesSaint-Paul-lez-Durance, France; 6 IRD, UMR Eco&SolsBâtiment 12, 2 Place Viala, F-34060, Montpellier Cedex 2, France

**Keywords:** Diversity, engineering, hydrogen metabolism, hydrogenase, oxygen

## Abstract

Hydrogen production by microorganisms is often described as a promising sustainable and clean energy source, but still faces several obstacles, which prevent practical application. Among them, oxygen sensitivity of hydrogenases represents one of the major limitations hampering the biotechnological implementation of photobiological production processes. Here, we describe a hierarchical biodiversity-based approach, including a chemochromic screening of hydrogenase activity of hundreds of bacterial strains collected from several ecosystems, followed by mass spectrometry measurements of hydrogenase activity of a selection of the H_2_-oxidizing bacterial strains identified during the screen. In all, 131 of 1266 strains, isolated from cereal rhizospheres and basins containing irradiating waste, were scored as H_2_-oxidizing bacteria, including *Pseudomonas* sp., *Serratia* sp., *Stenotrophomonas* sp., *Enterobacter* sp., *Rahnella* sp., *Burkholderia* sp., and *Ralstonia* sp. isolates. Four free-living N_2_-fixing bacteria harbored a high and oxygen-tolerant hydrogenase activity, which was not fully inhibited within entire cells up to 150–250 *μ*mol/L O_2_ concentration or within soluble protein extracts up to 25–30 *μ*mol/L. The only hydrogenase-related genes that we could reveal in these strains were of the *hyc* type (subunits of formate hydrogenlyase complex). The four free-living N_2_-fixing bacteria were closely related to *Enterobacter radicincitans* based on the sequences of four genes (16S rRNA, *rpoB*, *hsp60*, and *hycE* genes). These results should bring interesting prospects for microbial biohydrogen production and might have ecophysiological significance for bacterial adaptation to the oxic–anoxic interfaces in the rhizosphere.

## Introduction

Hydrogen gas (H_2_) naturally produced by fermentative biological processes, often termed as biohydrogen (BioH_2_), is a promising sustainable and clean energy vector (Das and Veziroglu 2001; Mudhoo et al. 2011). Hence, a substantial increase of the fermentative BioH_2_ production might help reducing the consumption of fossil fuels by replacing the common industrial methods for producing hydrogen, such as steam reformation of natural gas and coal gasification (Kotay and Das 2008). However, BioH_2_ production still remains difficult to achieve on a cost effective basis (Levin et al. 2004). Recent promising studies have focused on certain photosynthetic organisms (e.g., cyanobacteria and single-cell algae), which are able to produce hydrogen from water and sunlight (Das and Veziroglu 2001; Ghirardi et al. 2007). In the natural environment, this reaction takes place essentially in a transient manner in anaerobic conditions because the hydrogenases, which catalyze the reversible heterolytic cleavage of H_2_ (according to the reaction H_2_ ↔ H^−^ + H^+^ ↔ 2H^+^ + 2e^−^), are particularly sensitive to the presence of oxygen (O_2_) produced by oxygenic photosynthesis (Ghirardi et al. 2007). This limiting factor can account for the current low efficiency for photoproduction of BioH_2_ (Kalia and Purohit 2008).

Hydrogenases are found in Archaea and Bacteria, and in a lesser extent in Eukarya (Vignais et al. 2001). They are usually split into three classes: the [FeFe]-, the [NiFe]-, and the [Fe] hydrogenases, the latter showing a different mechanism as they catalyze hydride transfer to their cofactor methenyl-H_4_MPT^+^ and do not reduce artificial acceptors such as viologen dyes (Vogt et al. 2008). [FeFe]-hydrogenases, which actively evolve H_2_, are quickly and irreversibly inactivated in the presence of oxygen whereas most [NiFe]-hydrogenases are only reversibly inhibited by O_2_ and can be reactivated (Meyer 2007); [Fe]-hydrogenases are inactivated by O_2_ in cell extracts, but appear tolerant when purified (Shima and Thauer 2007). The molecular background for O_2_ sensitivity is not fully understood and represents one of the major limitations in BioH_2_ production processes. Limitations also include hydrogenase expression levels, electron-transfer rate limitations, and hindrances related to various regulatory/metabolic processes. Several strategies have been explored so far for biotechnological optimization of the photoproduction of BioH_2_. A first strategy consists of expressing oxygen-tolerant hydrogenases in photosynthetic organisms: this can be achieved by either expressing foreign O_2_-tolerant enzymes identified from bacterial diversity or by genetically modifying native enzymes based on known sequences of O_2_-tolerant hydrogenases (Burgdorf et al. 2005; Dementin et al. 2009). In this direction, targeted modification of residues forming a hydrophobic gas diffusion channel inside the hydrogenase was designed to limit O_2_ access to the active site and facilitate enzyme reactivation (Leroux et al. 2008; Liebgott et al. 2010). A second strategy consists of separating in time the phases of hydrogen production from those of oxygen production, by exploiting the metabolic flexibility of the organisms (Melis et al. 2000).

The first strategy can be enriched by the identification of new optimization pathways through surveys of the ecological diversity of hydrogen producing or oxidizing bacteria. In-depth ecological inventories of H_2_-producing/utilizing bacteria found in ecosystems known for accumulating hydrogen have been compiled in many studies. Unfortunately, few of these studies have fully characterized, at the molecular and functional levels, the hydrogenases involved in hydrogen reduction or oxidation in these ecosystems (Brito et al. 2005; Leul et al. 2005). Moreover, metagenomic studies have revealed that hydrogenase prevalence is not ubiquitous, suggesting that relatively few species are endowed with the ability to take up or evolve hydrogen (Meyer 2007). In addition, these hydrogenase-containing bacteria identified by metagenomics were found to be geographically restricted to few rich anaerobic niches, for example, vertebrate guts or bioreactor sludges (Meyer 2007). Searching for oxygen tolerance of hydrogenases should focus on targeted ecosystems having oxic–anoxic interfaces and accumulating hydrogen to substantial partial pressures, for example, hindgut periphery, aerated roots, biofilms, or soil aggregates (Brune et al. 2000).

Here, we report the setup and operation of a hydrogenase activity screening for hundreds of environmental bacterial strains collected from several ecosystems, as well as selection of bacterial isolates carrying oxygen-tolerant hydrogenases by mass spectrometry measurements. We finally focused our study on the characterization of oxygen-tolerant hydrogenases and of the strains that harbor these hydrogenases.

## Experimental Procedures

### Chemochromic screening of bacterial hydrogenases

This experimental assay was set up in order to measure an electroreduction occurring in the bacterial solutions in an H_2_ atmosphere by using the colorless oxidized methyl viologen (MV^2+^), which undergoes a reversible transition between oxidized MV^2+^ (colorless) and reduced MV^+^ (blue). Extended protocol details are provided in Supplementary Information and Figure S7. One thousand two hundred and sixty-six bacterial strains were screened from the CEA-LEMIRE collection (stored at −80°C). They comprised 667 isolates from a marine electroactive biofilm grown on a stainless steel cathode, 234 isolates from basins containing irradiating waste submerged in demineralized water (France) (Gales et al. 2004), 24 isolates from sewage sludges (France), 252 isolates from maize, wheat, and rice rhizospheres (originating from France, Vietnam, Egypt, and Senegal), and 89 isolates from vertisol microaggregates (Martinique).

### Characterization of the hydrogenotrophic bacteria

Total DNA of strains with active hydrogenase were extracted as recently described (Ranjard et al. 2003). Amplification of the 16S rRNA genes was performed by PCR (Achouak et al. 1999) using primers Fd1 and S17 (Haichar et al. 2007). Those primers enabled to obtain the nearly complete 16S rRNA gene sequences.

### Mass spectrometric measurements of gas exchange

Hydrogenase activities of 17 representative strains, selected as 1–3 strains from each bacterial genus (Table S1) were tested in vivo by mass spectrometric measurements of H_2_, HD, and D_2_ exchange, in both liquid and gas samples. In independent experiments, we tested the effect of formate on hydrogen production. Eventually, we tested whether *Enterobacter cloacae*/*Enterobacter radicincitans* strains were able to consume H_2_ in the presence of O_2_, by monitoring H_2_, O_2_, and CO_2_ exchange in cell suspensions at moderate O_2_ concentration after injection of H_2_. Extended protocols details are provided in Supplementary Information.

### Cell disruption and hydrogenase assays on soluble extracts

Bacteria were harvested by centrifugation and broken using a Constant cell disruption system (Constant Systems Ltd, U.K.). Membrane fractions were then discarded by ultracentrifugation and soluble, cell-free extract was used for hydrogenase assays (Gales et al. 2004). Extended protocol details are provided in Supplementary Information.

### Identification of the oxygen-tolerant hydrogenotrophic bacteria

Following ribosomal sequencing, a deeper identification was performed for strains belonging to the genus *Enterobacter* sp. Amplifications of *hsp*60 gene (primers Hsp60-F GGT AGA AGA AGG CGT GGT TGC and Hsp60-R ATG CAT TCG GTG GTG ATC ATC AG), the *groEL* homolog coding for the 60-kDa heat shock protein, and of *rpoB* gene coding for the RNA polymerase β subunit (primers RpoB-F AAC CAG TTC CGC GTT GGC CTG G and RpoB-R CCT GAA CAA CAC GCT CGG A), were also performed (Hoffmann and Roggenkamp 2003). Sequence data were obtained by single-pass double stranded analysis (Cogenics, Grenoble, France). The 16S rRNA, *rpoB*, and *hsp60* nucleotidic sequences of representative strains of the genus *Enterobacter* were obtained from GenBank. Basic Local Alignment Search Tool (BLAST) hits related to the *hycE* sequence from *Escherichia coli* SMS-3-5 were also downloaded from GenBank.

### Characterization of hydrogenase genes

Several approaches aiming at getting sequence information on the putative enzymes involved in bacterial hydrogen uptake were carried out, combining in silico searches of [NiFe] and [FeFe] hydrogenase sequence signatures, DNA amplifications using available universal primers and design of new degenerate primers. Extended protocols details are provided in Supplementary Information.

### Sequence data analysis

Sequences of the 16S rRNA, *rpoB*, *hsp60*, and *hycE* genes were aligned through the CLUSTAL-W software. Neighbor joining, including the Bootstrap Test of Phylogeny (10,000 replicates) using MEGA 4 (Tamura et al. 2007) was used.

### Nucleotide sequence accession numbers

The GenBank accession numbers for the sequences reported in this article are provided in Supplementary Information.

## Results

### Chemochromic screening of bacterial hydrogenases

By a methylviologen dye-based chemochromic screening of 1266 strains, we found 131 bacterial strains with positive response, that is, development of a blue color of the dye in anaerobic conditions under an H_2_ atmosphere. These strains were plated and identified using 16S rRNA gene sequencing. They belong to seven bacterial genera (Table S1), including 36 *Pseudomonas* sp., 34 *Serratia* sp., 23 *Stenotrophomonas* sp., 18 *Enterobacter* sp., 11 *Rahnella* sp., 6 *Burkholderia* sp., and 2 *Ralstonia* sp. isolates (Fig. S1). The majority of the positive strains were collected from basins containing irradiating waste (α, β, and γ emitting spent nuclear fuel) submerged in demineralized water. Strains of *Pseudomonas* sp., *Stenotrophomonas* sp., and *Serratia* sp. were the most represented ones in our sampling of cultivable bacteria collected from the basins. The remaining strains were isolated from maize, wheat, and rice rhizospheres (16 *Enterobacter* sp. and 2 *Stenotrophomonas* sp. isolates), and from vertisol microaggregates (two *Enterobacter* sp. isolates). No “positive” strains were collected from marine electroactive biofilms, in line with the view that environmental samples from oceans are probably not suited for identifying novel hydrogenases (Meyer 2007).

### Mass spectrometric measurements of gas exchange

**Figure 1:**
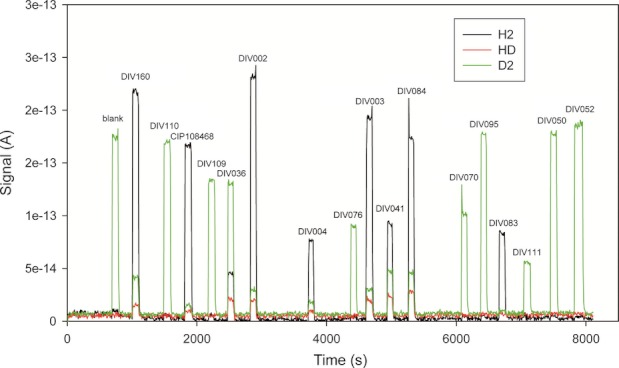
Isotope exchange in gas phase from cell-free buffer (blank) and a representative subset of 17 strains cultured in Hungate tubes in the presence of D_2_, selected as 1–3 strains from each bacterial genus. D_2_, H_2_, and HD signals were recorded by mass spectrometry. A high hydrogenase activity occurred with all the Enterobacteriaceae, including the strains identified as *Enterobacter* sp. (CIP108468, DIV036, DIV083, DIV084, and DIV160), *Rahnella* sp. (DIV041), and *Serratia* sp. (DIV002, DIV003, and DIV004) resulting in substantial H^+^/D_2_ exchange activity, as attested by significant increase in H_2_ or HD levels. All the strains shown in this figure were tested at least twice from independent cultures with this assay and gave similar results.

**Figure 2:**
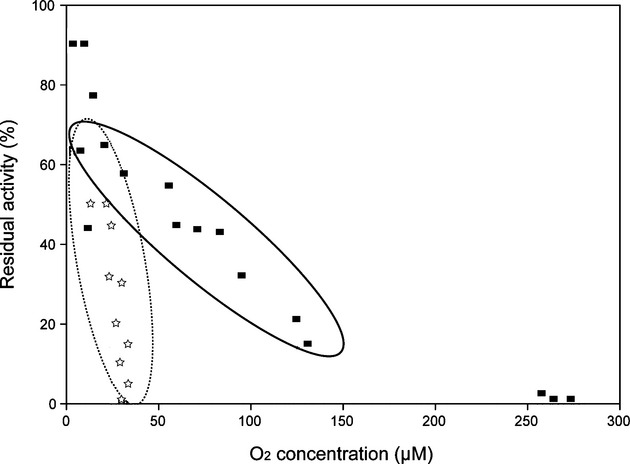
H^+^/D_2_ exchange activity of intact cells of the H_2_-oxidizing Enterobacteriaceae depending on the oxygen level of the spectrophotometer chamber. Each point represents an independent experiment, where a single strain was submitted to a given O_2_ level. The strains DIV002, DIV003, and DIV004 (*Serratia* sp.) and *Escherichia coli* (DIV001), which are inhibited at low concentration of O_2_ (dashed– dotted ellipse) are indicated with stars. In contrast, all the *Enterobacter cloacae* strains (DIV036, DIV140, DIV160, and DIV167), which are indicated with black dots, are only partially inhibited below ca. 150 *μ*mol/L O_2_ (solid ellipse).

The hydrogenase activity of 17 representative strains, selected as 1–3 strains from each bacterial genus (Table S1), was tested by their capacity to catalyze the H^+^/D^+^ scrambling reaction in presence of D_2_, which results in D_2_ consumption and in production of HD and H_2_. Two mass spectrometric methods based on liquid and gas samples were employed to measure H_2_/HD/D_2_ exchange. A high hydrogenase activity occurred with all the Enterobacteriaceae (nine strains, including the strains identified as *Enterobacter* sp., *Rahnella* sp., and *Serratia* sp.) resulting in substantial isotope exchange detectable in the gas phase of cultures performed in Hungate tubes (Fig. 1). For the other bacterial genera (eight strains), hydrogenase activity was much weaker, and only detectable in concentrated samples analyzed by membrane-inlet mass spectrometry (data not shown). We therefore decided to focus on strains from the Enterobacteriaceae family and tested how hydrogenase activity is affected by O_2_ exposure in vivo (Fig. S2A and B), using seven strains from our screen and *E. coli* as a common representative of this family. The hydrogenase activity of the majority of those Enterobacteriaceae strains was inhibited at low concentration of O_2_ (Fig. 2). However, the hydrogenase activity of four *Enterobacter* sp. strains (DIV036, DIV140, DIV160, and DIV167) was only partially inhibited by O_2_ up to 150 *μ*mol/L (air level being around 250 *μ*mol/L). These strains originate from different worldwide samplings of rhizospheres of *Poaceae* and, surprisingly, had all previously been identified as *E. cloacae* (Heulin et al. 1982; Omar et al. 1989; Berge et al. 1991). They were then selected for in-depth analysis of their hydrogenase activity and reactivity toward oxygen. Indeed, tolerance to oxygen in vivo might proceed from either hydrogenase enzyme tolerance or respiratory oxygen scavenging processes which might prevent enzyme exposure.

**Figure 3:**
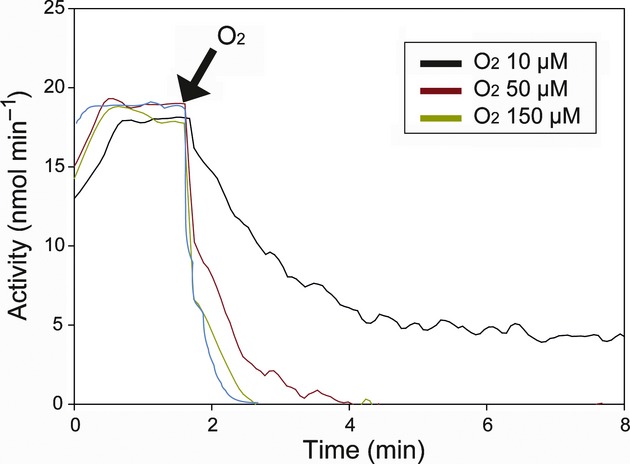
Hydrogenase activity within the soluble protein fraction extracted from the *Enterobacter radicincitans* DIV036 strain, depending on the oxygen level of the spectrophotometer chamber. Assays were conducted at 30°C, using different samples from the same extract, with total protein content adjusted to 2 mg/mL. The blue line indicates the hydrogenase activity of the highly oxygen-sensitive [NiFe] hydrogenase isolated from *Desulfovibrio fructosovorans* (purified enzyme, 0.1 *μ*g / mL), assayed in the same conditions and exposed to 10 *μ*mol / L O_2_.

**Figure 4:**
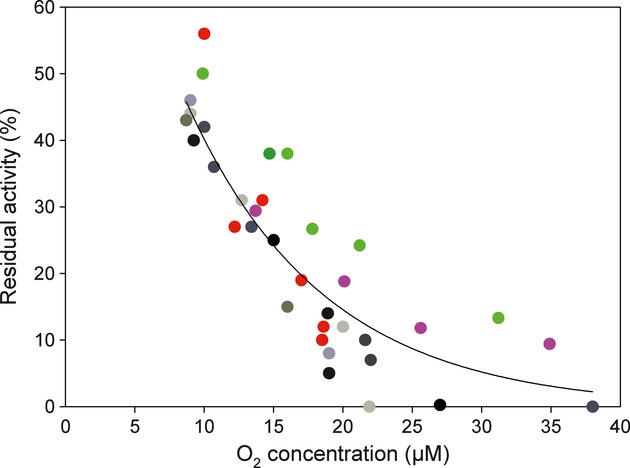
H^+^/D_2_ exchange activity within the soluble protein fraction extracted from the five *E. cloacae*/*E. radicincitans* strains (black: DIV036; red: DIV140; green: DIV160; dark green: DIV167; and purple: CIP108468) depending on the oxygen level in the mass spectrometer inlet chamber. On the same graph were also put measurements achieved in the same way on strains belonging to the DIV036-DIV140 cluster as seen in Figure S1 (DIV117, DIV118, DIV120, DIV121, DIV124, DIV125: successively represented using increasing shades of gray). Each point represents an independent experiment, where a single strain was submitted to a given O_2_ level.

Hydrogenase activity and reactivity toward O_2_ were further assessed ex vivo, within the soluble protein fraction of the *E. cloacae* cells. A typical set of experiments is shown in Figure 3: after injecting 10 *μ*mol/L of O_2_ to this fraction suspended in measurement buffer, the hydrogenase activity decreased down to approximately 25% of its initial level, whereas it was totally inhibited at 50 *μ*mol/L of O_2_. These results indicate that the isolated hydrogenase activity is also only partially inhibited at moderate concentrations of O_2_, which are however sufficient to fully inhibit O_2_ sensitive enzymes (Fig. 3, blue line). This means that at least one part of the O_2_-tolerance observed in vivo is due to the intrinsic properties of the hydrogenase enzyme. Hydrogenase activities of the protein fractions from the four *E. cloacae* strains, as well as those from isolates closely related to DIV036 and DIV140, showed similar O_2_ concentration responses (Fig. 4).

We subsequently checked whether *E. cloacae* (strain DIV036) was able to consume H_2_ in the presence of O_2_ in physiological conditions, by monitoring H_2_, O_2_, and CO_2_ exchange in cell suspensions at moderate O_2_ concentration (Fig. S3). In these conditions, both H_2_ uptake and transient stimulation of O_2_ uptake were observed following H_2_ injection. H_2_ uptake was clearly dependent on the presence of O_2_, as it was largely halted when O_2_ became exhausted (Fig. S3). In addition, we observed that in the middle and at the end of the H_2_ uptake period, the ratio of H_2_ to O_2_ consumption rates was close to 2:1, which is the expected stoichiometry in the case of simple electron transfer from H_2_ to O_2_ (Knallgas reaction). However, at the beginning of H_2_ exposure: both H_2_ and O_2_ uptake rates were greater – O_2_ uptake being much greater than expected from H_2_ consumption (Fig. S3). Interestingly, this phenomenon is correlated with a CO_2_ release peak (Fig. S3), which might indicate that H_2_ addition transiently stimulated the catabolism (decarboxylation) of some carbon substrate.

### Characterization of the oxygen-tolerant hydrogenotrophic bacteria

**Figure 5:**
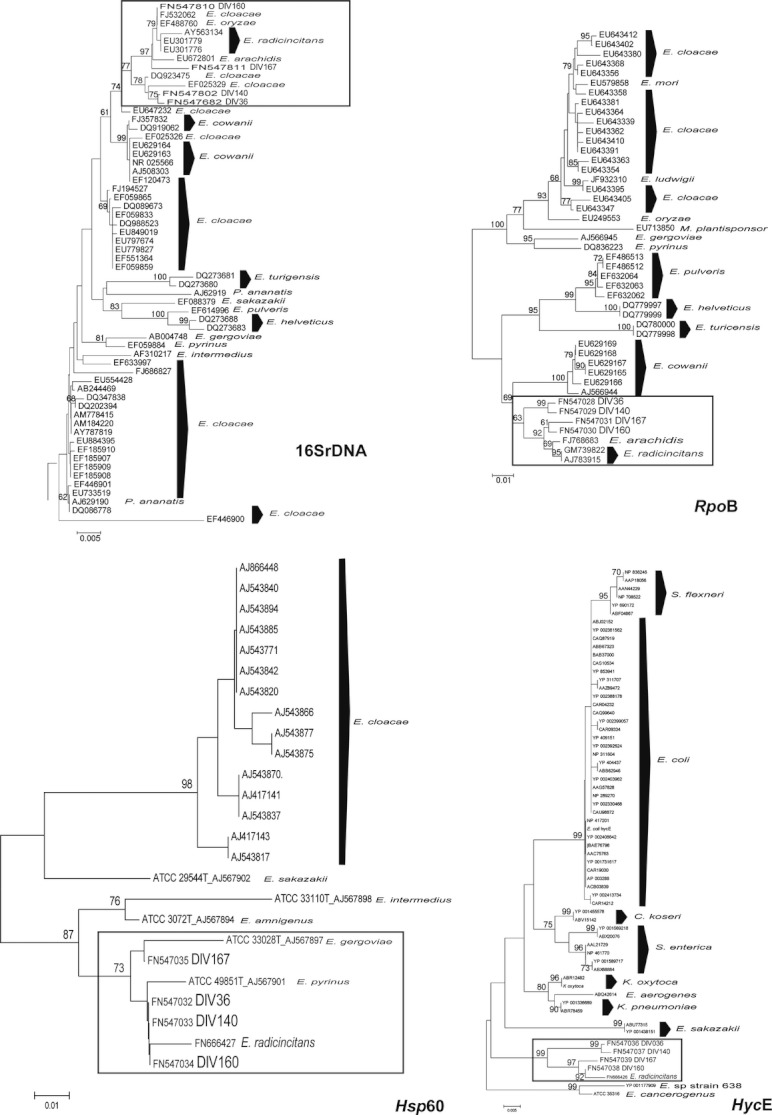
Phylogenetic neighbor joining trees with bootstraps value (above 70%) of 16S rRNA, *hsp60*, *rpoB,* and *hycE* partial sequences amplified from the H_2_-oxidizing *E. cloacae* strains collected from the cereal rhizospheres and partial sequences of other *Enterobacter* species available in GenBank.

A multiloci sequencing approach was applied in order to characterize the four *E. cloacae* strains isolated from the rhizosphere of rice and maize that were carrying an oxygen-tolerant hydrogenase (DIV036, DIV140, DIV160, and DIV167). The analyses of three housekeeping genes (16S rRNA, *hsp60*, and *rpoB*) were initially included in the study in order to look at the relationships between the four *E. cloacae* strains isolated from the rhizospheres of Poaceae and representative strains of *Enterobacter* sp., and see how these four *E. cloacae* cluster within the *Enterobacter* genus (Martens et al. 2008). The 16S rRNA genes from the four strains were closely related to those of *E. radicincitans*, *Enterobacter arachidis*, and *Enterobacter oryzae* (Peng et al. 2009), and to two sequences (accession numbers DQ923475 and FJ532062) amplified from *E. cloacae* strains associated with the rhizospheres of other Poaceae (*Eleusine indica* and *Chrysopogon zizanioides*). The *rpoB* sequences from the four strains were closely related to the *rpoB* sequences of *E. radicincitans* and of *E. arachidis* isolated from groundnut (Madhaiyan et al. 2010) (Fig. 5). The bootstrap values of the 16S rRNA and *rpoB* nodes grouping these strains were 91% and 77%, respectively. Strikingly, and not in agreement with previously published results (Peng et al. 2009), the *rpoB* sequence from *E. oryzae*, isolated from surface sterilized roots of wild rice *Oryza latifolia*, was weakly related (91% identity) to the four *rpoB* sequences of the *E. cloacae* strains and to the *rpoB* sequence of the type strain of *E. radicincitans*. The NJ tree obtained with the sequences of the *hsp60* gene indicates that the four *E. cloacae* strains clustered together with strains of *E. radicincitans, Enterobacter pyrinus*, and *Enterobacter gergoviae*. The four *E. cloacae* identified in this study and the type strain CIP108468 of *E. radicincitans* were split into two highly robust subgroups (DIV160/DIV167/CIP108468 vs. DIV036/DIV140) by analyzing the 16S rRNA and *rpoB* genes (bootstrap values ranging from 78% to 99%). Hydrogenase properties of the *E. radicincitans* type strain (CIP108468) were tested by mass spectrometric measurements in the same conditions as described above for the strains identified in this study. First, in gas phase H_2_/HD/D_2_ exchange assays (Hungate tubes), *E. radicincitans* CIP108468 exhibited a high activity (Fig. 1), indicating substantial hydrogenase amounts in this strain. We then tested hydrogenase reactivity toward O_2_ in soluble protein extracts of this strain. There again, the pattern of activity inhibition by O_2_ fitted well with the one observed in DIV160/167/036/140 (Fig. 4), indicating similar tolerance properties.

### Characterization of hydrogenase genes

A progressive hierarchical approach aiming at characterizing the enzymes putatively involved in the bacterial hydrogenase activity was carried out, combining in silico BLAST searches, DNA amplifications using available universal primers and design of degenerate primers. Regarding the in silico approach, neither homologs to *E. coli* [NiFe] hydrogenases 1, 2, and 4 nor to [FeFe] hydrogenases were detected by BLAST searches over the publicly available *E. cloacae* complete genomes: *E. cloacae* ssp. *cloacae* ATCC 13047 (Ren et al. 2010), *E. cloacae* EcWSU1 (Humann et al. 2011), *E. cloacae* ssp. *cloacae* NCTC 9394 (NCBI BioProject Ref PRJNA45967), and *E. cloacae* ssp. *dissolvens* SDM (Xu et al. 2012). The only hydrogenase operon detected within these genomes was the one corresponding to the *hyc* operon, which encodes the H_2_-evolving formate-hydrogenlyase (FHL) complex catalyzing the disproportionation of formate into CO_2_ and H_2_. The same features were observed for the genome of the closely related *Enterobacter* sp. 638, which interestingly is a plant growth-promoting endophytic bacterium, isolated from a poplar (*Populus trichocarpa* × *deltoides*) rhizome (Taghavi et al. 2009).

Furthermore, we could not amplify any DNA fragment from the DIV036, DIV140, DIV160, and DIV167 strains by using previously described universal degenerate primers of group 1 [NiFe] hydrogenase genes (Kim et al. 2007), whereas an expected 2.9–3 kb fragment, likely corresponding to Hyd1, was obtained from *E. coli* used as a control in the same conditions. In contrast, four pairs of degenerate primers used for amplifying fragments of *hycE* homologs (Table S2) successfully amplified fragments from these strains. These overlapping fragments were assembled, and the whole sequence of the *hycE* gene was obtained for the *E*. *cloacae* strain DIV036. Taken together, these results suggest that the *hyc* operon is likely to be the unique hydrogenase-encoding sequence present in the genome of the *E*. *cloacae* strains isolated from the roots of rice and maize. We have subsequently tested the FHL activity of *E. cloacae* (strain DIV036). The injection of formate into the chamber of the mass spectrometer has unambiguously induced H_2_ production in anaerobiosis (Fig. S4A and B).

Nondegenerated primers were then used for amplifying the whole sequence of the *hycE* gene from the four strains of *E*. *cloacae* (DIV036, DIV140, DIV160, and DIV167) and the type strain of *E. radicincitans* CIP108468 (Table S2). These primers were not totally specific to those five strains as faint bands were also obtained with other strains of *Enterobacter* sp. (DIV139, DIV141, DIV155, DIV156, and DIV158) isolated from cereal rhizospheres. The sequences of *hycE* obtained from the four strains of *E*. *cloacae* (DIV036, DIV140, DIV160, and DIV167) and from the type strain of *E. radicincitans* CIP108468 clustered together in a new branch within the *hycE* NJ phylogenetic tree, with a very high value of bootstrap of 99% (Fig. 5). Like in the phylogenetic trees built with housekeeping genes, the five strains were split into two highly robust subgroups (DIV160/DIV167/CIP108468 vs. DIV036/DIV140), with bootstraps values ranging from 97% to 99% (Fig. 5). The alignments of the five HycE sequences (DIV036, DIV140, DIV160, DIV167, and CIP108468) and the sequences from the most related Enterobacteriaceae showed pairwise scores (calculated as the number of identities in the best alignment divided by the number of the compared residues) ranging from 92% to 95% (Fig. S5). Ten residues were specific to the protein sequences of DIV036, DIV140, DIV160, DIV167, and CIP108468 when compared with the HycE sequences recovered by BLAST searches (alignment performed on more than 50 HycE sequences retrieved from databases) (Fig. S5).

## Discussion

### Basins containing irradiating waste and rhizospheres are ecosystems of choice for studying H_2_-oxidizing bacterial populations

We have used a biodiversity-based approach aiming at detecting hydrogenases from a collection of strains collected from various ecosystems worldwide, several of them being known for accumulating hydrogen to substantial partial pressures and harboring oxic–anoxic interfaces. A chemochromic screening was devised for recovering those strains harboring hydrogenases. The majority of the strains which positively responded to this screen were collected in two types of ecosystems, an industrial one – basins containing irradiating waste submerged in demineralized water, and an agricultural one – the rhizospheres of several cereals. These two ecosystems are known for producing molecular hydrogen, which is oxidized by their H_2_-oxidizing bacterial populations (Lechner and Conrad 1997; Gales et al. 2004). Moreover, it has been shown that aerotolerant, H_2_/CO_2_-dependent lifestyle and related enzymatic capacities occur in the rice rhizosphere (Erkel et al. 2006). Our chemochromic method confirmed the H_2_-oxidizing bacterial population richness of these two ecosystems. Hence, seven bacterial genera were identified in the basins and two in the rhizospheres. Novel genera of bacteria in which hydrogenase activity is identified, are here described, including *Stenotrophomonas* sp., *Rahnella* sp., and *Serratia* sp., which have not been previously reported for carrying such an activity. This result emphasizes the fact that the ecological strategy is probably a promising approach for obtaining novel bacterial taxa of interest by screening natural biodiversity.

The seven taxa isolated from the basins are likely to be pioneer colonizers that have an adaptive advantage regarding their capacity to be H_2_ oxidizers. The bacterial taxa, including *Pseudomonas* sp., *Ralstonia* sp., *Burkholderia* sp., and *Stenotrophomonas* sp. are also known to frequently inhabit ultrapure water systems (Kulakov et al. 2002). The multiple occurrence of the H_2_-oxidizing capability in several taxonomically unrelated bacteria collected in the same basins ecosystem stresses the need to characterize, in further studies, the diversity of the hydrogenases present in these bacteria. In an evolutionary perspective, it would be of particular interest to check whether the H_2_-oxidizing capability was independently acquired by these strains or resulted from lateral gene transfer (via local plasmid acquisition for instance). The presence of a plasmid involved in hydrogen metabolism has already been described in several bacterial species, including *Acidovorax* sp., *Pseudomonas* sp., and *Alcaligenes* sp. (Friedrich and Schwartz 1993; Lenz et al. 1997). Furthermore, the plasmid genes encoding the ability for the H_2_-oxidizing bacterium *Alcaligenes hydrogenophilus* to grow chemolithoautotrophically with H_2_ and CO_2_ were cloned in vivo and successfully transferred to other bacterial species (Miura and Umeda 1994).

### Oxygen-tolerant hydrogenases are carried by worldwide bacteria indigenous to rhizospheres of cereal crops

We have chosen to carry out an in-depth molecular characterization of bacteria that were harboring the most active and oxygen-tolerant hydrogenases in this set, that is, four *E. cloacae* strains isolated from rice and maize rhizospheres (Heulin et al. 1982; Omar et al. 1989; Berge et al. 1991). We have adopted a polyphasic approach, including the partial sequencing of three loci (16 rRNA, *hsp60*, and *rpoB*), known to reliably indicate genetic relationship among the *Enterobacter* species (Hoffmann and Roggenkamp 2003; Paauw et al. 2008), and a phenotypic characterization. This polyphasic approach unambiguously indicated that the four strains of *E*. *cloacae* (DIV036, DIV140, DIV160, and DIV167) were more closely related to the type strain of *E. radicincitans* CIP108468, which was originally described from a nitrogen- fixing and plant growth-promoting bacterial strain isolated from the rhizosphere of winter wheat (Kampfer et al. 2005). However, we cannot definitively state that the four strains of *E. cloacae* isolated from rice and maize belong to the *E. radicincitans* bacterial species, hence we have decided to keep their previous name (*E. cloacae*) along this study. These phylogenetic groupings were in line with previous results of phenotypic characterization (Fig. S6 and Table S3). The four phylogenetic trees also indicated that the four strains of *E*. *cloacae* (DIV036, DIV140, DIV160, and DIV167) and the type strain of *E. radicincitans* CIP108468 were always split into two subgroups, that is, DIV036 (rice, Egypt) clustered with DIV140 (maize, France) and DIV160 (rice, Senegal) clustered with DIV167 (rice, France) and CIP108468 (winter wheat, Germany), suggesting that no significant phylogeographical patterns occur within this bacterial taxon. Hence, those two subgroups are probably widespread and are not limited to geographically restricted regions. Seed exchanges and crop regular rotation should partly account for this geographical distribution of the strains. Rice and maize seeds have the potential to spread and transmit beneficial and deleterious bacteria, including *Enterobacter* species (Cottyn et al. 2001). In addition, a study has shown that *E. cloacae* strains are found in endophytic bacteria which are associated with the inner surface of the hull of rice seeds (Mukhopadhyay et al. 1996). Furthermore, in the Egyptian rice region, from which was isolated DIV036, rice usually follows maize in the regular rotation (Omar et al. 1989). These results suggest that the five *E. cloacae/E. radicincitans* strains have probably a “broad” host range and are able to colonize at least the maize, the rice, and the winter wheat rhizosphere, as well as the *Brassica oleracea* roots (Ruppel et al. 2006). The biology of the four strains described in our study should resemble the biology of the previously described *E. radicincitans* bacterium, which is able to successfully compete with the native bacterial community in plant tissues without inducing defense mechanisms and to enhance the growth of the host plant (Ruppel et al. 2006). These diazotrophic plant tissue-colonizing bacteria may offer an advantage as biofertilizer if their competitiveness was preserved in soil conditions (Ruppel et al. 2006).

### *HycE* from *E. radicincitans* is a reversible oxygen-tolerant hydrogenase

The most studied member of the Enterobacteriaceae, *E. coli*, possesses at least four hydrogenases (Vignais and Billoud 2007; Trchounian et al. 2012). Hydrogenases 1 and 2 are usually described as uptake hydrogenases, whereas hydrogenase 3, encoded by the *hycABCDEFGHI* genes, is reported to be a hydrogen-evolving enzyme (Vignais and Billoud 2007). However, recent studies have shown that hydrogenase 3 is a reversible hydrogenase that combines hydrogen uptake activity and hydrogen production (Maeda et al. 2007). We here report that the five strains of *E. cloacae*/*E. radicincitans* only possess *E. coli* hydrogenase 3 homologs, that also appear to be reversible, catalyzing H_2_ oxidation (in the presence of methylviologen) and H_2_ production (in anaerobiosis, in the presence of formate). Hydrogenase 3 is part of the FHL complex which couples formate dehydrogenase to proton reduction (Vignais and Billoud 2007). In *E. coli*, HycE (hydrogenase 3 large subunit) is only loosely attached to the other subunits of the FHL complex (Sauter et al. 1992), but the H_2_ oxidizing/benzylviologen reducing capacity of hydrogenase 3 is extremely labile and usually lost in soluble fraction or when attempting purification. Recently, Pinske et al. (2011) attributed this to the loss of connection between HycE and the FeS cluster bearing small subunit HycG, which is essential for electron transfer toward viologen acceptors. Indeed, at variance with HycE, HycG is tightly membrane bound (Sauter et al. 1992). Note that hydrogenases 1 and 2 which retain large/small subunits connectivity upon solubilization also retain viologen-reducing capacity (Pinske et al. 2011). In our case, activity is assessed by H/D exchange, which does not need FeS cluster connectivity, but only function of the NiFe active site. This might be one of the reasons why we have access to activity of HycE in soluble fraction. Another possibility might be that the case is different in *E. cloacae*, and that the hydrogenase moiety of the FHL complex keeps associated in the soluble fraction. It was even recently reported that a Hyd3 complex could be purified from *Klebsiella oxytoca* and was oxygen tolerant (Wu et al. 2011).

The five *E. cloacae*/*E. radicincitans* strains, isolated from cereal rhizospheres, showed similar levels of inhibition by oxygen of their hydrogenase activity (Figs. 2 and 4). In this study, we have obtained the whole sequence of the *hycE* gene. Ten residues are specific to the *E. cloacae*/*E. radicincitans hycE* genes that we described, including three leucine/methionine substitutions, when comparing their sequences to those of other enterobacterial *hycE*, including the *hycE* gene from the oxygen-tolerant *K. oxytoca* HP1 (Wu et al. 2011). The hydrogenase activity measured with the entire cells and within the soluble protein fraction is completely inhibited only when the amount of injected O_2_ ranged from 150 to 250 *μ*mol/L and from 25 to 30 *μ*mol/L, respectively. The other Enterobacteriaceae selected by the chemochromic test were all highly sensitive to oxygen and immediately inhibited when small amounts of O_2_ (i.e., <10 *μ*mol/L) were injected. Nevertheless, the molecular modifications involved in this “oxygen tolerance” remain unknown. The recent release of the whole genome of a Rice Cluster-I methanogenic archaeon had shed light on a combination of unique sets of antioxidant enzymes and DNA repair as well as oxygen-insensitive enzymes allowing the archaea to outcompete other methanogens in their habitats (Erkel et al. 2006). It remains unclear whether those ten residues may be significant for oxygen reactivity. Indeed, the oxygen tolerance may be due to multiple molecular modifications located in different parts of the FHL complex, or involve factors encoded by different parts of the bacterial genome. In addition, we observed concomitant H_2_ and O_2_ uptake at moderate O_2_ concentration. Electron transfer from H_2_ to O_2_ (Knallgas reaction) is not expected to occur in relation with hydrogenase 3 function, as this enzyme is not supposed to feed electrons into the respiratory chain through quinone reduction. Our observation might be due to either an indirect connection trough metabolic intermediates or to an *E. cloacae*-specific mechanism remaining to be resolved. In the same experiment, H_2_ addition also transiently stimulated the catabolism (decarboxylation) of a carbon substrate. Similar results were reported for the nitrogen-fixing bacterium *Azotobacter vinelandii*: H_2_ addition into the bacterial cultures significantly stimulated the mannose utilization and consequently the bacterial growth (Wong and Maier 1985). The ability to aerobically use H_2_ might therefore be a means of facilitating degradation of otherwise recalcitrant carbon substrates.

Our results are in-line with the finding that endophytic *E. cloacae* isolated from rice seeds are able to express (at least partly) their hydrogenase in an aerobic environment (Mukhopadhyay et al. 1996). Although developing in an often flooded and therefore anoxic environment, rice roots receive oxygen through the aerenchyma of the plant, favoring the development of oxic–anoxic interfaces. Oxygenation of the rhizosphere is heterogeneous, and steep oxygen gradients are formed along the rice roots (Brune et al. 2000). Revsbech et al. (1999) have shown that the oxygen saturation inside a rice root of a 3-week-old rice transplant at 8.5-cm distance from the base is constant and equal to 52% of air saturation, that is, approximately 125 *μ*mol/L. This value is in the range of the threshold of total inhibition of the hydrogenase activity in vivo that we report in this study, suggesting that the hydrogenase of *E. cloacae*/*E. radincincitans* strains developing in this area would not be fully inhibited, even if its activity would be dramatically decreased. This highlights the fact that bacteria that are living near the rice roots, or even inside the root, may have been constrained to adapt their metabolism for facing oxygenation of the rice rhizosphere (Revsbech et al. 1999). Moreover, a remaining activity of the hydrogenase for a weak O_2_ saturation of the rhizosphere, and/or a quick recovery of the activity after a total inhibition of the enzyme by high O_2_ saturation could be an evolutionary adaptation allowing an optimal life within the soil influenced by the rice roots.

This might help sustaining an “oxygen-tolerant” hydrogen metabolism, which is likely to provide *E. cloacae*/*E. radicincitans* a selective advantage (for instance: contribute to N_2_ fixation through intracellular O_2_ scavenging, or help in metabolizing recalcitrant substrates such as those issued from plant decomposition) that might contribute to their prevalence in worldwide rice rhizosphere.

## Supporting Information

**Table S1.** List of the strains with positive response, that is, development of a blue color of the dye in anaerobic conditions under an H_2_ atmosphere (H_2_-oxidizers) by using the chemochromic screening method. In the “Mass spectrometry columns,” we indicate the strains that were further tested by measuring their hydrogenase activity using two mass spectrometric methods based on hydrogen isotope assays in liquid or gas samplings.

**Table S2.** Pairs of degenerate primers (pairs 1–4) and nondegenerate primers (pair 5) for amplifying fragments of the *hycE* gene and parts of the flanking genes.

**Table S3.** Biochemical and physiological tests. Results which are divergent among the strains are highlighted as follows: orange corresponds to a divergence between the group of strains constituted by *Enterobacter radicincitans*, DIV036, DIV140, DIV160, DIV167, and E. cowani or *E. arachidis* or *E. oryzae* and green corresponds to variable results among the DIV036, DIV140, DIV160, and DIV167 strains.

**Figure S1.** Phylogenetic neighbor joining tree with boot-straps value (above 70%) of 16S rRNA partial sequences amplified from H_2_-oxidizing strains collected from the basins containing irradiating waste and the cereal rhizospheres.

**Figure S2.** (A) H_2_/HD/D_2_ exchange activity of intact cells from the *Enterobacter radicincitans* DIV036 strain. The chamber was saturated with D_2_ before being manually closed. H_2_ and HD production started as soon as the cells were in anoxia. Oxygen (80 *μ*mol/L) was added 5 min after the closure of the chamber. HD and H_2_ production subsequently dropped down after this O_2_ injection. When the medium became anoxic again, hydrogenase activity recovered and HD/H_2_ production resumed. (B) Time course monitoring of hydrogenase activity calculated from the rates of H_2_ and HD production, which were obtained from derivation of the gas concentrations in panel A over time. With an O_2_ injection of 80 *μ*mol/L, the activity was not absolutely disrupted and was maintained at the level of approximately 2.5 nmol/min mg/prot, while O_2_ decreased again due to the aerobic respiration of the *Enterobacter cloacae* strain. Hydrogenase activity progressively recovered after O_2_ was completely removed by respiration. All gas concentrations are expressed in *μ*mol/L.

**Figure S3.** H_2_, O_2_, and CO_2_ exchange after injection (indicated by arrow) of H_2_ at moderate O_2_ concentration, monitored by membrane-inlet mass spectrometry, in *Enterobacte cloacae* cells. Rates of O_2_ and H_2_ uptake have been calculated and are indicated above corresponding curves at given time points.

**Figure S4.** Effect of formate addition on O_2_ consumption and H_2_ production, monitored by membrane-inlet mass spectrometry, in *Enterobacter cloacae* cells previously resuspended in phosphate buffer. (A) Formate addition in aerobic conditions stimulated respiration, H_2_ production was observed once anaerobiosis was reached. (B) Formate addition in anaerobic conditions stimulates H_2_ production. Both experiments are indicative of formate-hydrogenlyase activity.

**Figure S5.** Aligned protein sequences of HycE from the five *E. cloacae*/*E. radicincitans* strains collected from the cereal rhizospheres and from closely related Enterobacteriaceae whose sequences were downloaded from GenBank. Amino acid changes between sequences are highlighted: red color corresponds to an *E. cloacae*/*E. radicincitans*-specific mutation, the blue color corresponds to an *E. cloacae*/*E. radicincitans* subgroup-specific residue, and the yellow color corresponds to an *E. cloacae*/*E. radicincitans* strain-specific residue. Ten residues are specific to the protein sequences of the five *E. cloacae*/*E. radicincitans* strains, they are located at the following positions: 17 (K for *E. cloacae*/*E. radicincitans* vs. A for the other Enterobacteriaceae), position 72 (F vs. Y), 97 (L vs. N/D), 207 (V vs. I), 213 (M vs. L), 337 (M vs. L), 357 (M/T vs. Q), 364 (M vs. L), 439 (L vs. Y/F), and 560 (G vs. S). Twelve additional residues are specific to either a subgroup of the *E. cloacae*/*E. radicincitans* strains or to a single *E. cloacae*/*E. radicincitans* strain (strain specific).

**Figure S6.** Dendrogram showing phenotypic similarities among isolates from this study, *E. cloacae*/*E. radicincitans*, *Enterobacter oryzae*, and *Enterobacter cowanii*. The results listed in Table S3 were converted into a binary matrix. The similarity matrix was computed with Jaccard coefficient, and the Unweighted Pair Group Method with Arithmetic mean (UPGMA) was used for cluster analysis (DendroUPGMA; available from: http://genomes.urv.es/UPGMA/).

**Figure S7.** Procedures of the chemochromic screening of bacterial hydrogenases.
