# Supplementary material for: Reversible oxygen-tolerant hydrogenase carried by free-living N2-fixing bacteria isolated from the rhizospheres of rice, maize, and wheat
Source: Microbiologyopen. 2012 Sep 12;1(4):349–61. doi: 10.1002/mbo3.37 (PMC3535381; doi:10.1002/mbo3.37)
Supplement: Supplementary file 1 [file mbo30001-0349-SD8.doc]

Table S1.

List of the strains with positive response, i.e. development of a blue colour of the dye in anaerobic conditions under an H_2_ atmosphere (H_2_-oxidizers) by using the chemochromic screening method. In the “Mass spectrometry columns”, we indicate the strains that were further tested by measuring their hydrogenase activity using two mass spectrometric methods based on hydrogen isotope assays in liquid or gas samplings.

| **Strain** | **Other Number** | **Organism** | **Origin** | **Year** | **Mass spectrometry** | |
| --- | --- | --- | --- | --- | --- | --- |
|  |  |  |  |  | ***Gaz sample*** | ***Liquid sample*** |
| CIP108468 | D5/23 | *E. radicincitans* | Institut Pasteur’s Collection |  | **√** | **√** |
| DIV001 | Dh5alpha | *E. coli* |  |  | **√** | **√** |
| DIV002 | G1 | *Serratia* sp. | Basins containing irradiating waste | 2003 | **√** | **√** |
| DIV003 | G7 | *Serratia* sp. | ‘‘ | 2003 | **√** | **√** |
| DIV004 | 004 | *Serratia* sp. | ‘‘ | 2003 | **√** | **√** |
| DIV006 |  | *Stenotrophomonas* sp. | ‘‘ |  |  |  |
| DIV007 |  | *Stenotrophomonas* sp. | ‘‘ |  |  |  |
| DIV008 |  | *Pseudomonas* sp. | ‘‘ | 1995 |  |  |
| DIV009 |  | *Pseudomonas* sp. | ‘‘ |  |  |  |
| DIV010 |  | *Rahnella* sp. | ‘‘ |  |  |  |
| DIV011 |  | *Stenotrophomonas* sp. | ‘‘ | 2002 |  |  |
| DIV012 |  | *Pseudomonas* sp. | ‘‘ | 2001 |  | **√** |
| DIV013 |  | *Serratia* sp. | ‘‘ | 2001 |  |  |
| DIV014 |  | *Pseudomonas* sp. | ‘‘ |  |  |  |
| DIV015 | F1 | *Serratia* sp. | ‘‘ | 2003 |  |  |
| DIV016 |  | *Pseudomonas* sp. | ‘‘ |  |  |  |
| DIV017 |  | *Pseudomonas* sp. | ‘‘ |  |  |  |
| DIV018 | J2 | *Serratia* sp. | ‘‘ | 2001 |  |  |
| DIV019 |  | *Pseudomonas* sp. | ‘‘ |  |  |  |
| DIV020 |  | *Pseudomonas* sp. | ‘‘ |  |  |  |
| DIV021 |  | *Pseudomonas* sp. | ‘‘ |  |  |  |
| DIV022 |  | *Stenotrophomonas* sp. | ‘‘ |  |  |  |
| DIV023 |  | *Serratia* sp. | ‘‘ | 2003 |  |  |
| DIV024 |  | *Serratia* sp. | ‘‘ | 2003 |  |  |
| DIV025 |  | *Serratia* sp. | ‘‘ | 2003 |  |  |
| DIV026 |  | *Serratia* sp. | ‘‘ | 2003 |  |  |
| DIV027 |  | *Serratia* sp. | ‘‘ | 2003 |  |  |
| DIV028 |  | *Serratia* sp. | ‘‘ | 2003 |  |  |
| DIV029 |  | *Serratia* sp. | ‘‘ | 2001 |  |  |
| DIV030 |  | *Serratia* sp. | ‘‘ | 2001 |  |  |
| DIV031 |  | *Serratia* sp. | ‘‘ | 2003 |  |  |
| DIV032 |  | *Serratia* sp. | ‘‘ | 2003 |  |  |
| DIV033 |  | *Stenotrophomonas* sp. | Rhizosphere of rice (Vietnam) | 1998 |  |  |
| DIV034 |  | *Serratia* sp. | Basins containing irradiating waste | 2003 |  |  |
| DIV035 |  | *Serratia* sp. | ‘‘ | 2003 |  |  |
| DIV036 | NO9 | Enterobacter sp. | Rhizosphere of rice (Egypt) | 1983 | **√** | **√** |
| DIV037 | G9 | *Serratia* sp. | Basins containing irradiating waste | 2003 |  |  |
| DIV038 | F3 | *Serratia* sp. | ‘‘ | 2003 |  |  |
| DIV039 | F2 | *Serratia* sp. | ‘‘ | 2003 |  |  |
| DIV040 |  | *Rahnella* sp. | ‘‘ |  |  |  |
| DIV041 |  | *Rahnella* sp. | ‘‘ |  | **√** | **√** |
| DIV042 |  | *Stenotrophomonas* sp. | ‘‘ |  |  |  |
| DIV043 |  | *Serratia* sp. | ‘‘ | 2003 |  |  |
| DIV045 |  | *Rahnella* sp. | ‘‘ | 2003 |  |  |
| DIV046 |  | *Stenotrophomonas* sp. | ‘‘ |  |  |  |
| DIV047 |  | *Stenotrophomonas* sp. | ‘‘ |  |  |  |
| DIV048 |  | *Stenotrophomonas* sp. | ‘‘ |  |  |  |
| DIV049 |  | *Stenotrophomonas* sp. | ‘‘ |  |  |  |
| DIV050 |  | *Stenotrophomonas* sp. | ‘‘ |  | **√** |  |
| DIV051 |  | *Stenotrophomonas* sp. | ‘‘ |  |  |  |
| DIV052 |  | *Pseudomonas* sp. | ‘‘ | 1995 | **√** | **√** |
| DIV053 |  | *Pseudomonas* sp. | ‘‘ | 1995 |  |  |
| DIV054 |  | *Pseudomonas* sp. | ‘‘ | 1995 |  |  |
| DIV055 |  | *Pseudomonas* sp. | ‘‘ | 1995 |  |  |
| DIV056 |  | *Pseudomonas* sp. | ‘‘ |  |  |  |
| DIV057 |  | *Pseudomonas* sp. | ‘‘ |  |  |  |
| DIV058 |  | *Pseudomonas* sp. | ‘‘ |  |  |  |
| DIV059 |  | *Serratia* sp. | ‘‘ | 2003 |  |  |
| DIV060 |  | *Pseudomonas* sp. | ‘‘ | 2003 |  |  |
| DIV061 |  | *Pseudomonas* sp. | ‘‘ |  |  |  |
| DIV062 |  | *Pseudomonas* sp. | ‘‘ |  |  |  |
| DIV063 |  | *Burkholderia* sp. | ‘‘ | 2001 |  |  |
| DIV064 |  | *Burkholderia* sp. | ‘‘ | 2001 |  |  |
| DIV065 |  | *Serratia* sp. | ‘‘ | 2003 |  |  |
| DIV066 |  | *Serratia* sp. | ‘‘ | 2003 |  |  |
| DIV067 |  | *Pseudomonas* sp. | ‘‘ | 1995 |  |  |
| DIV068 |  | *Pseudomonas* sp. | ‘‘ | 1995 |  |  |
| DIV069 |  | *Pseudomonas* sp. | ‘‘ | 1995 |  |  |
| DIV070 |  | *Pseudomonas* sp. | ‘‘ |  | **√** | **√** |
| DIV071 |  | *Pseudomonas* sp. | ‘‘ |  |  |  |
| DIV072 |  | *Pseudomonas* sp. | ‘‘ | 2001 |  | **√** |
| DIV073 |  | *Serratia* sp. | ‘‘ | 2001 |  |  |
| DIV074 |  | *Stenotrophomonas* sp. | ‘‘ |  |  |  |
| DIV075 |  | *Stenotrophomonas* sp. | ‘‘ |  |  |  |
| DIV076 |  | *Pseudomonas* sp. | ‘‘ | 1995 | **√** | **√** |
| DIV077 |  | *Pseudomonas* sp. | ‘‘ | 1995 |  |  |
| DIV078 |  | *Serratia* sp. | ‘‘ | 2001 |  |  |
| DIV079 |  | *Serratia* sp. | ‘‘ | 2003 |  |  |
| DIV080 |  | *Pseudomonas* sp. | ‘‘ |  |  |  |
| DIV081 |  | *Pseudomonas* sp. | ‘‘ |  |  |  |
| DIV082 |  | *Serratia* sp. | ‘‘ | 2003 |  |  |
| DIV083 | M10 | *Enterobacter* sp. | Vertisol microaggregates |  | **√** | **√** |
| DIV084 | M9 | *Enterobacter* sp. | ‘‘ |  | **√** | **√** |
| DIV085 |  | *Serratia* sp. | Basins containing irradiating waste | 2003 |  |  |
| DIV086 |  | *Serratia* sp. | ‘‘ | 2001 |  |  |
| DIV087 |  | *Serratia* sp. | ‘‘ | 2001 |  |  |
| DIV088 |  | *Serratia* sp. | ‘‘ | 2003 |  |  |
| DIV089 |  | *Serratia* sp. | ‘‘ | 2003 |  |  |
| DIV090 |  | *Stenotrophomonas* sp. | Rhizosphere of rice (Vietnam) | 1997 |  |  |
| DIV091 |  | *Pseudomonas* sp. | Basins containing irradiating waste |  |  |  |
| DIV092 |  | *Pseudomonas* sp. | ‘‘ |  |  |  |
| DIV093 |  | *Pseudomonas* sp. | ‘‘ |  |  |  |
| DIV094 |  | *Pseudomonas* sp. | ‘‘ |  |  |  |
| DIV095 |  | *Stenotrophomonas* sp. | Rhizosphere of rice (Vietnam) | 1997 | **√** |  |
| DIV096 |  | *Rahnella* sp. | Basins containing irradiating waste |  |  |  |
| DIV097 |  | *Rahnella* sp. | ‘‘ |  |  |  |
| DIV098 |  | *Pseudomonas* sp. | ‘‘ | 1995 |  |  |
| DIV099 |  | *Pseudomonas* sp. | ‘‘ | 1995 |  |  |
| DIV100 |  | *Stenotrophomonas* sp. | ‘‘ | 1995 |  |  |
| DIV101 |  | *Stenotrophomonas* sp. | ‘‘ |  |  |  |
| DIV102 |  | *Stenotrophomonas* sp. | ‘‘ |  |  |  |
| DIV103 |  | *Stenotrophomonas* sp. | ‘‘ |  |  |  |
| DIV104 |  | *Stenotrophomonas* sp. | ‘‘ |  |  |  |
| DIV105 |  | *Stenotrophomonas* sp. | Rhizosphere of rice (Vietnam) | 1997 |  |  |
| DIV106 |  | *Stenotrophomonas* sp. | ‘‘ | 1997 |  |  |
| DIV107 |  | *Pseudomonas* sp. | Basins containing irradiating waste |  |  |  |
| DIV108 |  | *Burkholderia* sp. | ‘‘ |  |  |  |
| DIV109 |  | *Burkholderia* sp. | ‘‘ |  | **√** | **√** |
| DIV110 |  | *Ralstonia* sp. | ‘‘ |  | **√** | **√** |
| DIV111 |  | *Ralstonia* sp. | ‘‘ | 2003 | **√** | **√** |
| DIV112 |  | *Burkholderia* sp. | ‘‘ | 2001 |  |  |
| DIV113 |  | *Burkholderia* sp. | ‘‘ | 2001 |  |  |
| DIV117 | NO42 | *Enterobacter* sp. | Rhizosphere of rice (Egypt) | 1983 |  | **√** |
| DIV118 | NO12 | *Enterobacter* sp. | ‘‘ | 1983 |  | **√** |
| DIV120 | NO4 | *Enterobac ter* sp. | ‘‘ | 1983 |  | **√** |
| DIV121 | NO29 | *Enterobacter* sp. | ‘‘ | 1983 |  | **√** |
| DIV124 | NO3 | *Enterobacter* sp. | ‘‘ | 1983 |  | **√** |
| DIV125 | NO31 | *Enterobacter* sp. | ‘‘ | 1983 |  | **√** |
| DIV136 | CF261 | *Rahnella* sp. | Rhizosphere of wheat (France) | 1986 |  | **√** |
| DIV137 | CF211 | *Rahnella* sp. | ‘‘ | 1986 |  | **√** |
| DIV138 | PS10 | *Rahnella* sp. | Rhizosphere of maize (France) | 1986 |  | **√** |
| DIV139 | PS11 | *Enterobacter* sp. | ‘‘ | 1986 |  | **√** |
| DIV140 | PS12 | *Enterobacter* sp. | ‘‘ | 1986 |  | **√** |
| DIV141 | PS13 | *Enterobacter* sp. | ‘‘ | 1986 |  | **√** |
| DIV146 | CF8 | *Rahnella* sp. | Rhizosphere of wheat (France) | 1986 |  | **√** |
| DIV154 | CF101 | *Rahnella* sp. | ‘‘ | 1986 |  | **√** |
| DIV155 | 15ATR | *Enterobacter* sp. | Rhizosphere of rice (France) | 1975 |  | **√** |
| DIV156 | 7ATR | *Enterobacter* sp. | ‘‘ | 1975 |  | **√** |
| DIV158 | L33 | *Enterobacter* sp. | ‘‘ | 1982 |  | **√** |
| DIV159 | CIP6085 | *Enterobacter* sp. | Spinal fluid | 1978 |  | **√** |
| DIV160 | B1 | *Enterobacter* sp. | Rhizosphere of rice (Senegal) | 1977 | **√** | **√** |
| DIV167 |  | *Enterobacter* sp. | Rhizosphere of rice (France) | 1975 |  | **√** |
