# Supplementary material for: Reversible oxygen-tolerant hydrogenase carried by free-living N2-fixing bacteria isolated from the rhizospheres of rice, maize, and wheat
Source: Microbiologyopen. 2012 Sep 12;1(4):349–61. doi: 10.1002/mbo3.37 (PMC3535381; doi:10.1002/mbo3.37)
Supplement: Supplementary file 2 [file mbo30001-0349-SD9.doc]

Table S2.

Pairs of degenerate primers (pairs 1-4) and non-degenerate primers (pair 5) for amplifying fragments the whole *hycE* gene and parts of the flanking genes.

| **Primer** | **Pair**  **(product size)** | **Sequence** | **Orientation** | **Gene**  **(*E*. sp. 638)** | **Position** |
| --- | --- | --- | --- | --- | --- |
| Entero2For | 1 (1029 bp) | TCGTT(A/G)ATGCGAACTTTCAG | Forward | Ent638_3194 | 401-420 |
| Entero2Rev | 1 (1029 bp) | GGA(T/C)(C/G)CGAATAAACCGGA(A/G)T | Reverse | Ent638_3194 | 1410-1429 |
| EnteroHyd3For1 | 2 (1566 bp) | TTTACGCAGCGGGTAGAGTT | Forward | Ent638_3194 | 1261-1280 |
| EnteroHyd3Rev1 | 2 (1566 bp) | GAACG(G/C)TT(T/C)GGCATCAAAGT | Reverse | Ent638_3192 | 554-573 |
| CloacaeFor4 | 3 (380 bp) | TTTACGCAGCGGGTAGAGTT | Forward | Ent638_3194 | 1261-1280 |
| CloacaeRev4 | 3 (380 bp) | A(T/A)GA(A/G)(G/T)CCTGGCA(A/G)ACCAAA | Reverse | Ent638_3194 | 1621-1640 |
| CloacaeFor5 | 4 (482 bp) | AATCGGCACCACATTGTTTT | Forward | Ent638_3194 | 1159-1178 |
| CloacaeRev4 | 4 (482 bp) | A(T/A)GA(A/G)(G/T)CCTGGCA(A/G)ACCAAA | Reverse | Ent638_3194 | 1621-1640 |
| Hyd3largesuFor2 | 5 (2172 bp) | AAATCGGCTTTGTTCCACAC | Forward | Ent638_3193 | 227-246 |
| Hyd3largesuRev2 | 5 (2172 bp) | GCCCTGTTTGAGAACAGCAT | Reverse | Ent638_3195 | 107-126 |
