# Supplementary material for: Reversible oxygen-tolerant hydrogenase carried by free-living N2-fixing bacteria isolated from the rhizospheres of rice, maize, and wheat
Source: Microbiologyopen. 2012 Sep 12;1(4):349–61. doi: 10.1002/mbo3.37 (PMC3535381; doi:10.1002/mbo3.37)
Supplement: Supplementary file 3 [file mbo30001-0349-SD10.doc]

Table S3.

Biochemical and physiological tests. Results which are divergent among the strains are highlighted as follow: orange corresponds to a divergence between the group of strains constituted by *Enterobacter radicincitans*, DIV036, DIV140, DIV160, DIV167 and *E. cowani* or *E. arachidis* or *E. oryzae* and green corresponds to variable results among the DIV036, DIV140, DIV160 and DIV167 strains*.*

| Test | *E.radicincitans* | *E. arachidis* | *E. cowanii* | *E. oryzae* | DIV036 | DIV0140 | DIV0160 | DIV0166 |
| --- | --- | --- | --- | --- | --- | --- | --- | --- |
| Host | Phyllosphere  winter wheat | Rhizosphere  Groundnut | Human blood |  | Rhizosphere  rice | Rhizosphere  maize | Rhizosphere  rice | Rhizosphere  rice |
| Country | Germany | India | Japan | China | Egypt | France | Senegal | France |
| Region | Munich | Tamilnadu |  |  | Gisa | Landes | Sedhiou | Camargues |
| Gram | - | - | - |  | - | - | - | - |
| Anaeroby | Facultative |  | Facultative |  | Facultative | Facultative | Facultative | Facultative |
| Color of colonies | Beige |  | Yellow-White |  | Beige | Beige | Beige | Beige |
| GC % |  | 53.2 | 52.5-53.6 | 55.0±0.4 |  |  | 53 |  |
|  |  |  |  |  |  |  |  |  |
| Voges-Proskauer 37°C | + | + | + | + | + | + | + | + |
| Motility | + | + | + | + | + | + | + | + |
| Peritrichous flagella |  |  | + |  | + | + | + | + |
| Beta-D-galactosidase (ONPG) | + |  | + | + | + | + | + | + |
| Cytochrome oxydase | - | Weakly positive | - | - | - | - | - | - |
| Catalase | + | + | + |  | + | + | + | + |
| Arginine dihydrolase | + | + | - | + | + | + | + | + |
|  |  |  |  |  |  |  |  |  |
| Adonitol | - | + | - | + | - | - | - | - |
| L-Arabinose | + | + | + | + | + | + | + | + |
| D-Arabinose | + | + |  | - |  |  |  | + |
| Cellobiose | + | + | + | + | + | + | + | + |
|  |  |  |  |  |  |  |  |  |
|  |  |  |  |  |  |  |  |  |
| Test | *E.radicincitans* | *E. arachidis* | *E. cowanii* | *E. oryzae* | DIV036 | DIV0140 | DIV0160 | DIV0166 |
| Dulcitol | + | + | + | + | - | - | + | + |
| Esculin | + | - | + | - | + | + | + | + |
| D-Glucose | + | + | + | + | + | + | + | + |
| Glycerol |  | + | + | + | - | - | + | + |
| Inositol | - | + | - | - | - | - | - | - |
| Lactose | + | + | + | - | - | + | - | - |
| Maltose | + | + | + | + | + | + | + | + |
| D-Mannitol | + | + | + | + | + | + | + | + |
| α-Melibiose | - | - | + | + | + | - | - | - |
| Raffinose | - | + | + | + | + | - | - | - |
| L-Rhamnose | + | + | + | - | + | + | + | + |
|  |  |  |  |  |  |  |  |  |
| Salicin | + |  | + | - | + | + | + | + |
| Saccharose | + | + | + | + | + | + | + | + |
| D-Sorbitol | + | + | + | + | + | + | + | + |
| Sucrose | + | + | + | + | + | + | + | + |
| Trehalose | + | + | + | + | + | + | + | + |
| Xylose | + |  | + | + | + | + | + | + |
|  |  |  |  |  |  |  |  |  |
| Citrate | + | + | + | + | + | + | + | + |
| Mucate | - | - | + | + | + | + | + | + |
| Galacturonate |  |  |  | + | + |  | - | + |
|  |  |  |  |  |  |  |  |  |
| Lysine decarboxylase | - | - | - | + | - | - | - | - |
| Tryptophan deaminase | - |  |  | - | - | - | - | - |
| Urease | - | - | - | - | - | - | - | - |
| Indole | - | - |  | - | - | - |  | - |
|  |  |  |  |  |  |  |  |  |
| Malonate | + | + | - | - | + | + | - | + |
| Gelatinase |  | - | - | - | + | - | - | + |
| Ornithine decarboxylase | - | + | - | + | - | - | - | - |
| Nitrate reductase |  |  |  | - | + | + | + | + |
