# Supplementary figures and images for: Reversible oxygen-tolerant hydrogenase carried by free-living N2-fixing bacteria isolated from the rhizospheres of rice, maize, and wheat
Source: Microbiologyopen. 2012 Sep 12;1(4):349–61. doi: 10.1002/mbo3.37 (PMC3535381; doi:10.1002/mbo3.37)

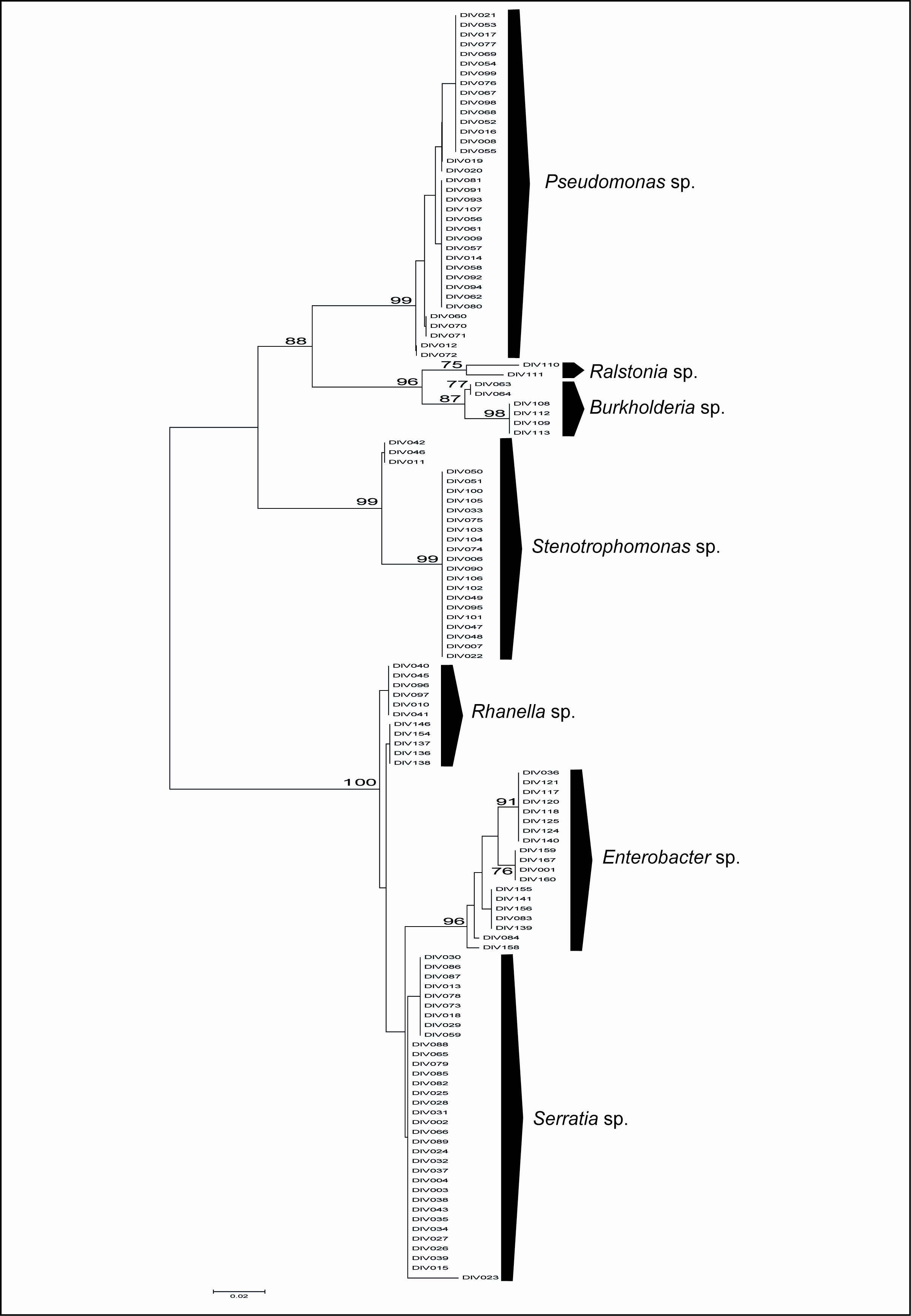

Supplement: Supplementary file 4 [file mbo30001-0349-SD1.tif]

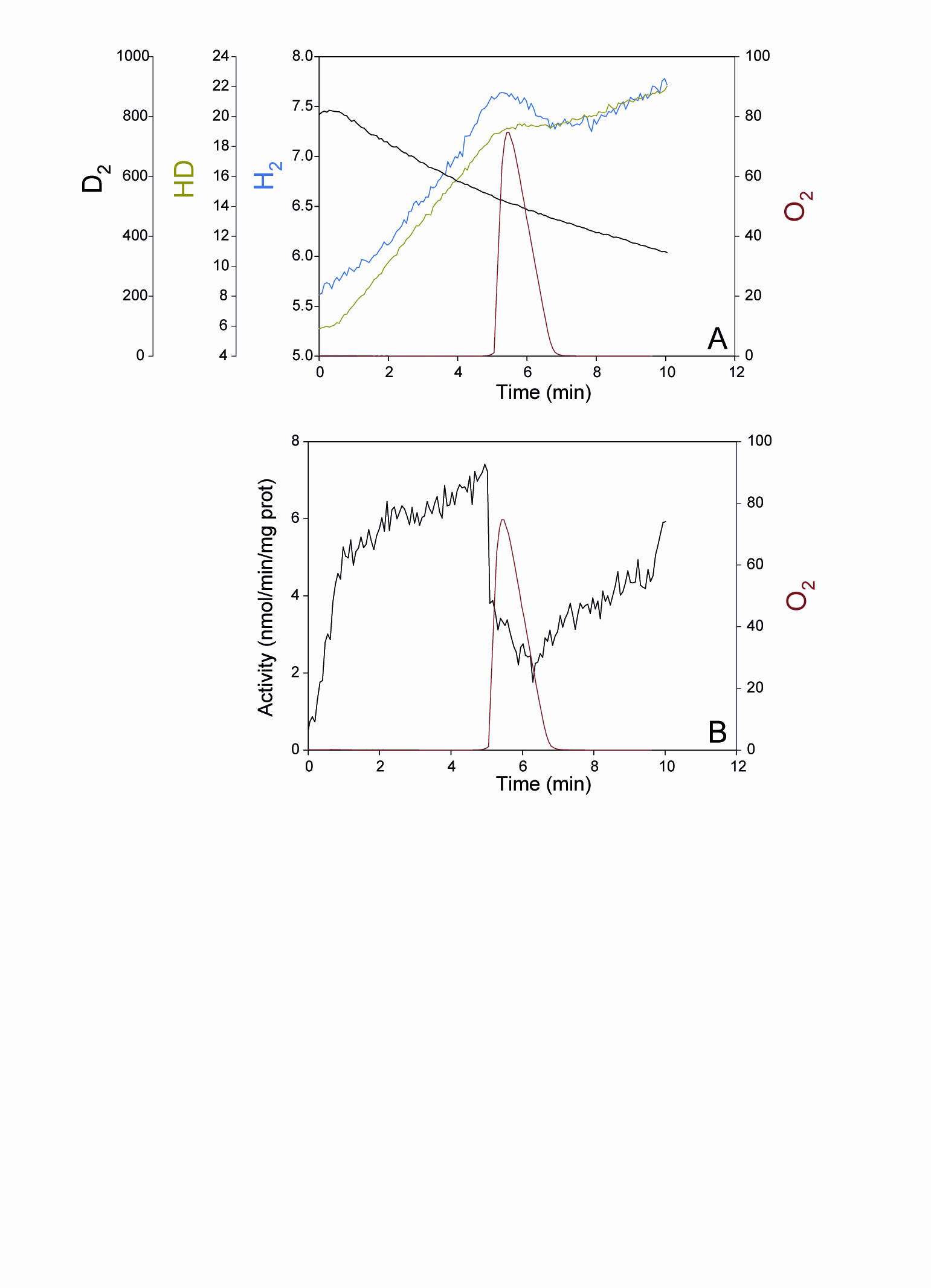

Supplement: Supplementary file 5 [file mbo30001-0349-SD2.tif]

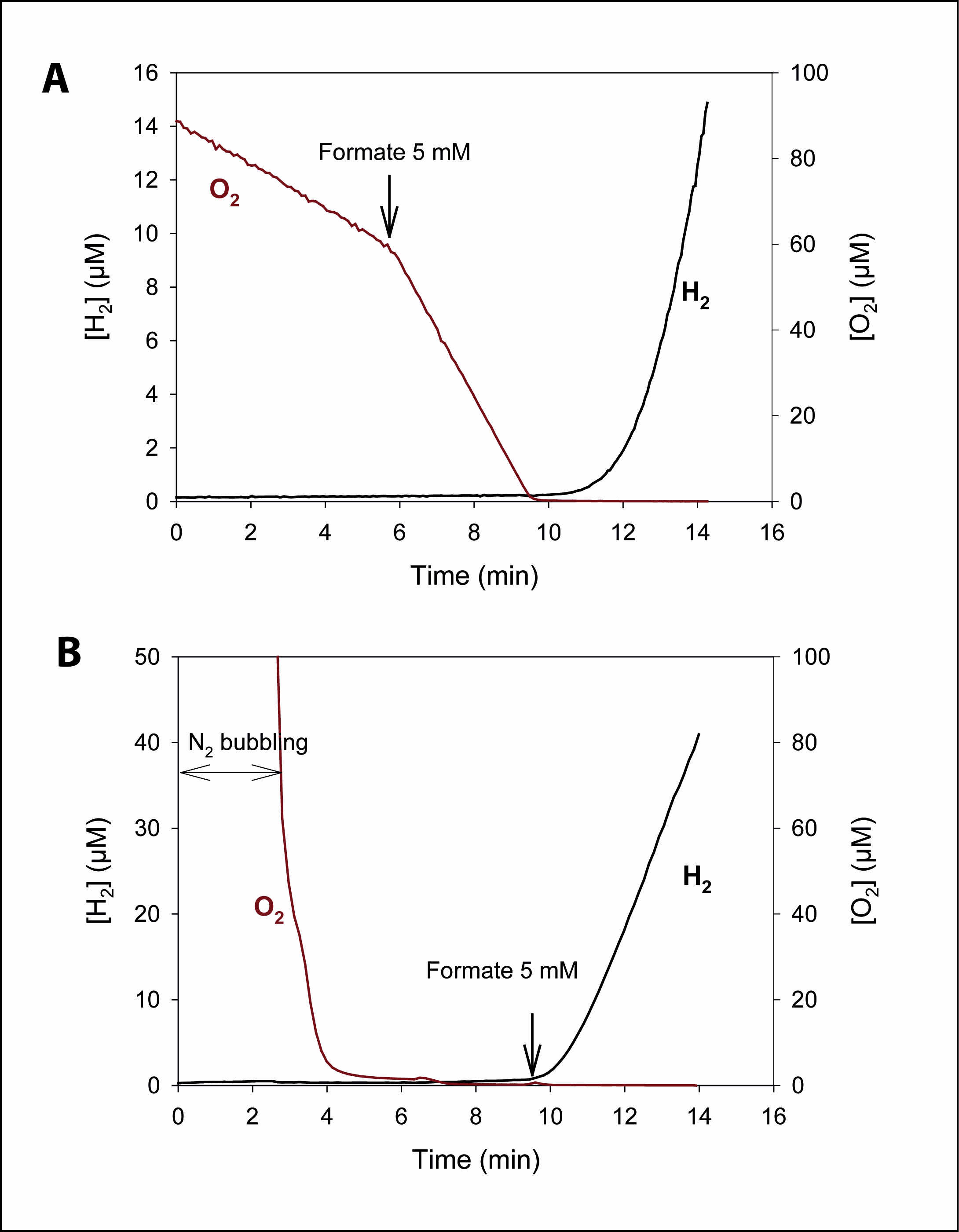

Supplement: Supplementary file 7 [file mbo30001-0349-SD4.tif]

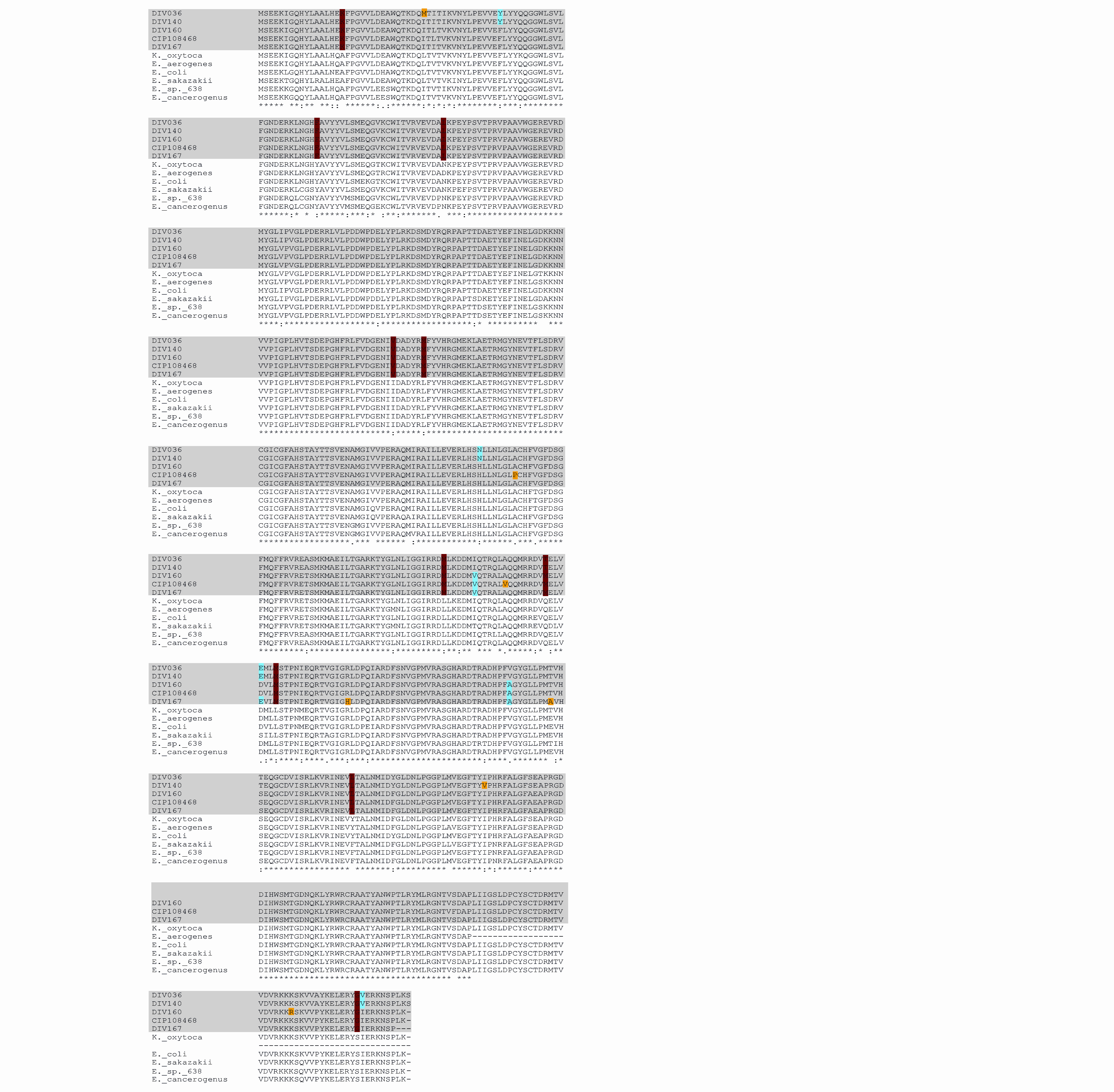

Supplement: Supplementary file 8 [file mbo30001-0349-SD5.tif]

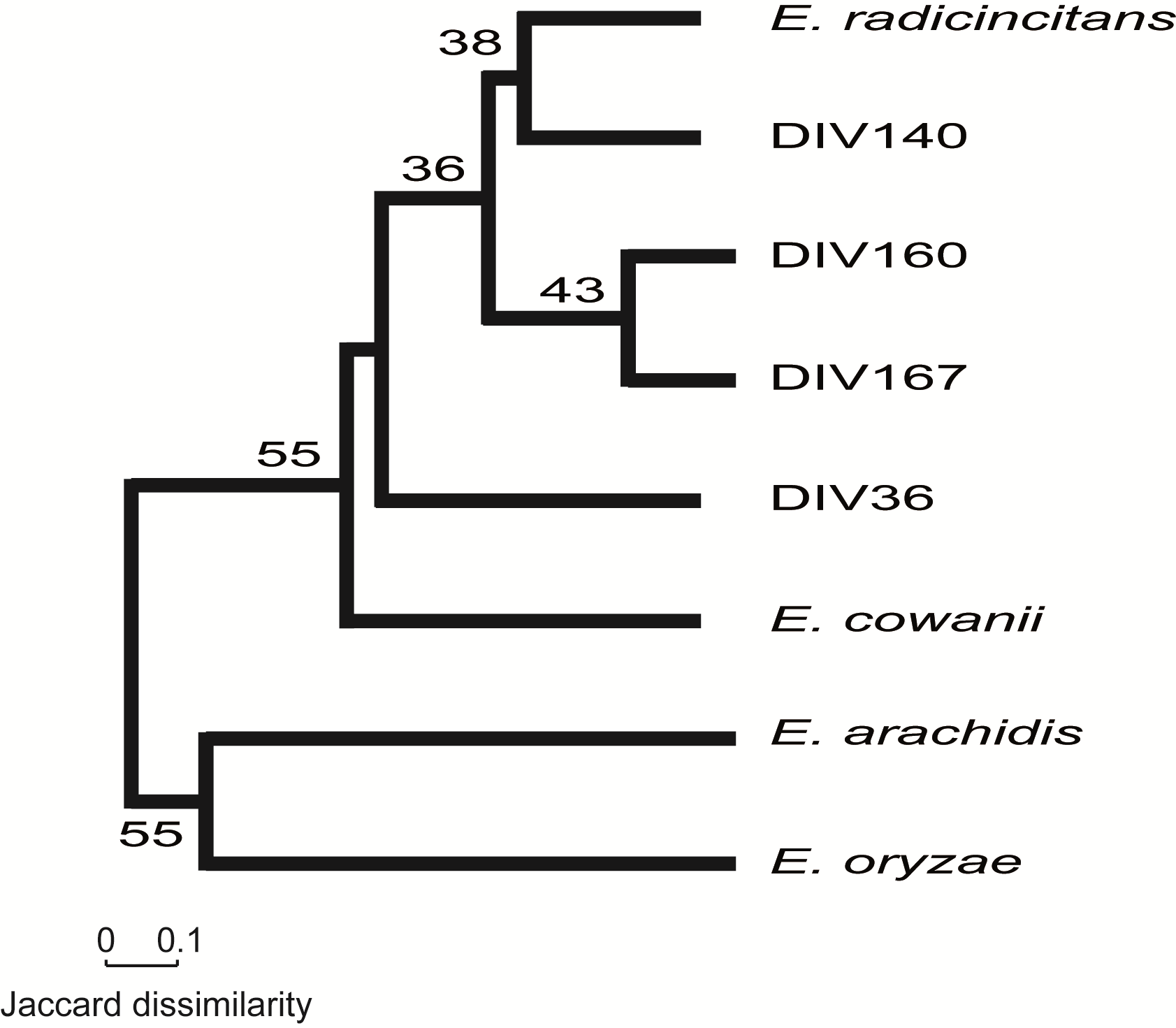

Supplement: Supplementary file 9 [file mbo30001-0349-SD6.tif]

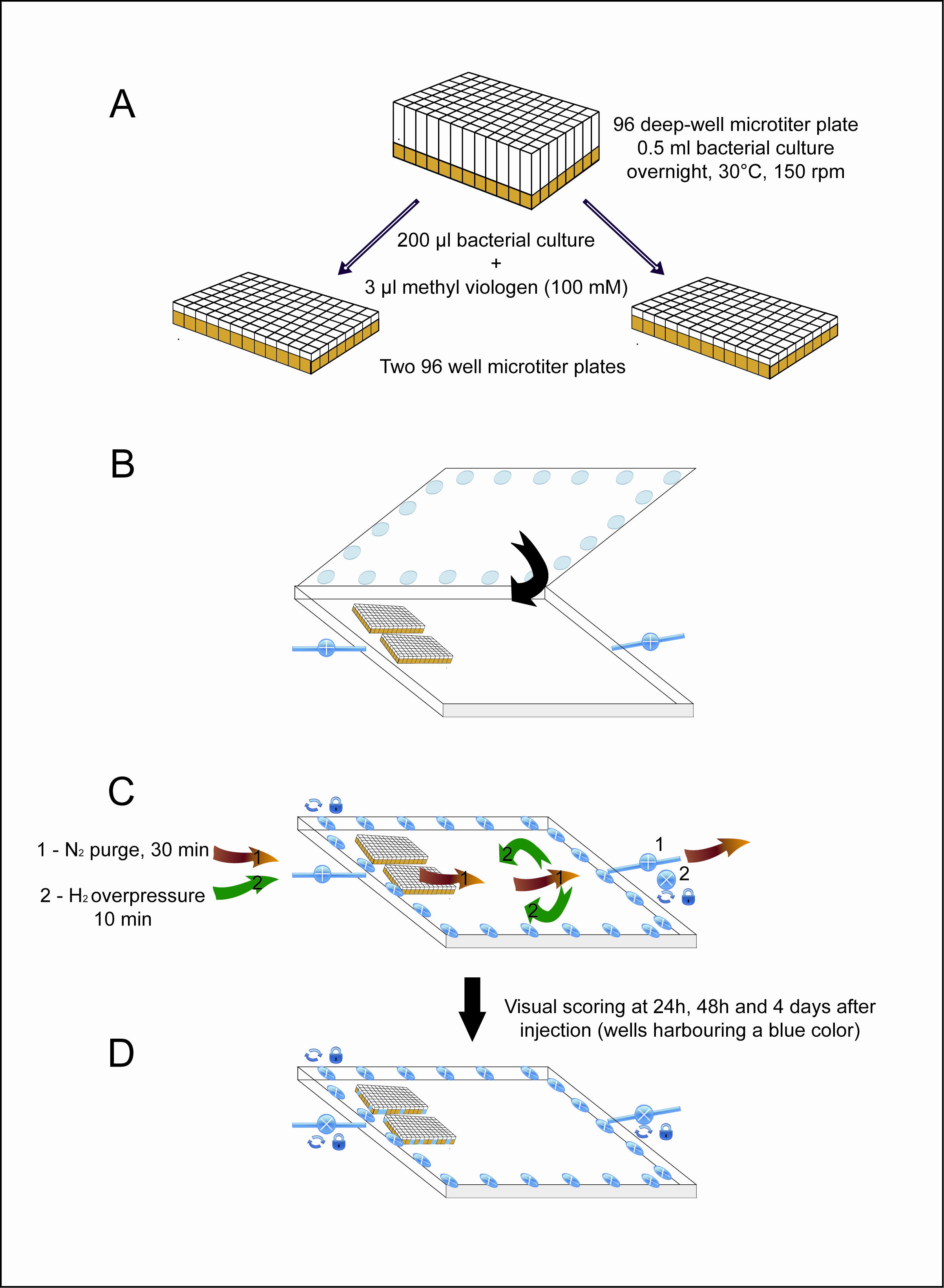

Supplement: Supplementary file 10 [file mbo30001-0349-SD7.tif]
